# Lessons from a feasibility study testing an anticipatory care planning intervention for older adults at risk of functional decline: feedback from implementing stakeholders

**DOI:** 10.1186/s40814-022-00973-w

**Published:** 2022-01-19

**Authors:** Dagmar Anna S. Corry, Gillian Carter, Frank Doyle, Kieran McGlade, Peter O’Halloran, Emma Wallace, Kevin Brazil

**Affiliations:** 1 Centre for Evidence and Social Innovation, Queen’s University Belfast, Belfast, Northern Ireland UK; 2 School of Nursing and Midwifery, Queen’s University Belfast, Belfast, Northern Ireland UK; 3 Department of Health Psychology, Royal College of Surgeons in Ireland, Dublin, Republic of Ireland; 4 School of Medicine, Dentistry, and Biomedical Sciences, Queen’s University Belfast, Dunluce Health Centre, Belfast, Northern Ireland UK; 5 Department of General Practice, RCSI University of Medicine and Health Sciences, Dublin, Republic of Ireland

**Keywords:** Anticipatory care planning, General practitioners, Pharmacists, Nurses, Person-centered, Older adults, Functional decline, Process of implementation, Primary care intervention

## Abstract

**Background:**

Anticipatory care is becoming increasingly important in effectively managing complex multimorbidity in aging populations, preventing further functional decline, and avoiding hospital admissions. This study aimed to elicit the feedback of participating general practitioners, practice managers, nurses and an adjunct pharmacist on the implementation strengths and limitations of a nurse-led, person-centered anticipatory care planning (ACP) intervention for older people at risk of functional decline in a primary care setting. The findings have implications for a full trial and intervention design.

**Methods:**

As part of a feasibility cluster randomized controlled trial (cRCT) testing the ACP intervention, we sought feedback from implementing stakeholders: general practitioners (*N* = 3), practice staff (*N* = 3), research nurses (*N* = 5), and adjunct pharmacist (*N* = 1) in both the Republic of Ireland (ROI) and Northern Ireland (NI), UK. Following written, informed consent, they were interviewed to investigate their experience of participating in the implementation of the ACP intervention as part of the feasibility trial, and elicit any recommendations for a full trial. Using the Consolidated Framework for Implementation Research, thematic analysis was employed to analyze data. The intervention consisted of home visits by specially trained nurses who assessed participants’ health, discussed with them their health goals and plans, and devised an anticipatory care plan following consultation with participants’ General Practitioners and the adjunct clinical pharmacist.

**Results:**

Participating stakeholders indicated that the strengths of the implementation process included the training provided to the nurses, constructive collaboration of the research team, and structure of implementation process. Perceived limitations included the selection process and screening tool, communication between the research team and the nurses, the assessment questionnaire, and the final document left with the patient, as well as lack of access to medical records for the adjunct pharmacist. Recommendations include better communication and team-wide consensus on alterations to procedure and documents, and standardized protocols for patient selection, data collection, and reporting for research nurses.

**Conclusions:**

The findings have identified strengths of the implementation process on which to build, and recognized limitations which can now be addressed to ensure improved efficiency and effectiveness in future trials.

**Trial registration:**

Clinicaltrials.gov, ID: NCT03902743. Registered on 4 April 2019.

**Supplementary Information:**

The online version contains supplementary material available at 10.1186/s40814-022-00973-w.

## Key messages regarding feasibility

- What uncertainties existed regarding the feasibility?

We interviewed the health professionals who implemented the intervention to obtain clarification as to which components of the intervention, and which areas of the implementation process need to be changed and improved in order to ensure an effective full trial.

- What are the key feasibility findings?

In order to improve the implementation process of this intervention in a full trial we garnered the views of the implementing stakeholders. Key findings of these interviews include employing standardized procedures and protocols that clarify and govern (1) data protection, (2) access to patient records, (3) effective communication, (4) screening, (5) assessment, and (6) uniform recording of research nurses’ data. Findings from the interviews have further helped to carve out the characteristics of the role of the anticipatory care nurse, and the supporting infrastructure required to make this a feasible and sustainable primary care based ACP intervention.

- What are the implications of the feasibility findings for the design of the main study?

The lessons from the feasibility study have direct implications for informing a full trial, similar studies, and similar interventions. If this intervention were to be upscaled the lessons gleaned from the feasibility study indicate that changes are required both at systemic level as well as at implementing team level (intervention characteristics, characteristics of individuals involved, process of implementation) to ensure effectiveness and sustainability of the intervention.

## Background

Two thirds of people aged 65 or over have two or more long-term health conditions [1] otherwise known as multimorbidity. Their healthcare is complicated due to the complex interactions of their conditions, medication regime and physiological changes associated with ageing. Management of multimorbidity is challenging as clinical guidelines tend to be single condition-oriented rather than person-oriented [2, 3] and patients with multimorbidity are under-represented in clinical trials. This can often lead to fragmented care and polypharmacy, with a detrimental effect on patients’ quality of life, increased treatment burden and increased use of health services [4].. With these patients making up around one third of general practice consultations, anticipatory care planning (ACP) interventions are increasingly important to improve quality of life, avoid hospital admissions, and to reduce polypharmacy and multiple healthcare appointments [5–8].

Health services on the island of Ireland have begun to move towards preventative, primary-care based, person-centered interventions [9, 10]. Managing people with multiple conditions safely and effectively in primary care depends on a well-organized and robust primary care system, which is usually defined as one that offers comprehensive care over a period of time to a defined population. This means that patients’ care can be followed up over time, chronic diseases monitored and managed, and different problems can be dealt with by the same team, thus integrating care around the patient [11]. We know that gaps between evidence and practice are commonplace in health care. Handley et al. [12] discuss how these gaps are often due to a lack of effort in identifying the factors for successful implementation of a particular intervention.

Against this background, we conducted a feasibility cluster randomized controlled trial (cRCT), examining and evaluating an anticipatory care planning intervention for older people at risk of functional decline [13]. As part of the evaluative process, we interviewed participating patients [14], key health professionals [15], and implementing stakeholders (ISH) about their views on the intervention. This paper focuses on the evaluation of the implementation process using interview data from the implementing stakeholders (general practitioners, practice managers, research nurses, and adjunct pharmacist). This will allow, in the spirit of transparency and transferability, valuable lessons from the feasibility study to be shared, and to facilitate optimization of the implementation strategy. Together this will ensure both best practice and outcomes for a full trial, and similar studies and interventions.

The Consolidated Framework for Advancing Implementation Research (CFIR) [16] guided this evaluation. While the focus is on the implementation process, we share all findings from the ISH interviews to provide the required context in terms of intervention characteristics, inner and outer settings, and characteristics of the individuals involved [16]. In sharing what we have learnt, we aim to lay the foundations for best practice guidelines for future efforts. We report perceived barriers and facilitators to the intervention itself, to the implementation process, providing recommendations for improvement where possible.

## Method

With the ACP protocol described in detail elsewhere [13] this paper focuses on the feedback from implementing stakeholders (ISH) about their experience with the process of implementing the intervention. They were participating GPs (*n* = 3) and practice managers (*n* = 3) (intervention arm), specifically trained research nurses (*n* = 5), and an adjunct pharmacist (*n* = 1). The paper follows COREQ guidelines for reporting qualitative research [17] (see Additional file 1) and the TIDieR checklist [18] (see Additional file 2). The CFIR [16] guided the evaluation, more specifically its component ‘process of implementation’ (PI) which includes planning, engaging, executing, and reflecting and evaluating.

Thematic analysis [19] was employed to examine feedback regarding the implementation process of the nurse-led ACP intervention for older adults at risk of functional decline. This involved qualitative interviews with 12 ISH to elicit their feedback regarding the implementation process in a bottom-up approach to establish its viability for a full trial. Three general practitioners (GP) and three GP practice managers (PM) were interviewed face-to-face on their own premises, with one practice in NI declining to be interviewed. The adjunct pharmacist was interviewed by telephone, four research nurses (RN) were interviewed at their place of work, and one at her home.

Prior to interview ISHs received a detailed written brief and information on the nature of the intervention, and provided written consent. Interviews were on average 30-min long, were audio recorded with a digital recorder, transcribed verbatim, and were accompanied by field notes.

### The intervention

The aim of the ACP intervention was to identify unrecognized problems among older (≥ 70 years) adults living in the community at increased risk of functional decline, and to develop a personalized support plan. A feasibility cluster randomized controlled trial was conducted by assigning eight GP practices to either the intervention or control arm (four per group) respectively. Practices were stratified by jurisdiction and by rurality prior to randomization. GP computerized clinical record systems were searched to identify eligible participants and, in line with NICE guidelines [20] the PRISMA-7 screening tool [21] was used to screen for risk of functional decline (see [13] for full protocol) for inclusion. PRISMA-7 is considered a best-practice tool to identify patients at risk of frailty in general practice, with those obtaining a score of ≥ 3 recognized as being at increased risk [22, 23]. All patients meeting the inclusion criteria (*n* = 73) were approached to participate and 65 were recruited. Allocation to the intervention (*n* = 34) or control group (*n* = 29) was conducted on a practice basis.

Preceding the home visits research nurses (*n* = 5) from both jurisdictions completed a three-day training program which was facilitated by a clinician expert in the field, and included study overview, principles and practice of personalized care, shared decision making, conducting of a personalized, holistic assessment with the EASY-Care [24] tool, and completing a medication review in collaboration with an adjunct clinical pharmacist.

Those in the intervention group received up to three home visits over 10 weeks (up to two hours in duration) by specially trained research nurses who assessed their physical, mental, and social health with the EASY-Care assessment tool [24], and discussed with them their health goals and plans for the purpose of drawing up a personalized support plan. Participants who did not require all three home visits received follow-up telephone calls. The ACP assessment, using the EASY-Care assessment tool, was conducted with the aid of a medical summary provided by the GP practice, including details of the patient’s health conditions and prescribed medications. Following that the nurse had a consultation with the participants’ GPs and the adjunct clinical pharmacist who conducted a medication review, and through this liaison the final personalized care plan was developed. Both patient and GP were provided with a copy of the final ACP.

### Sample

Eight GP practices were recruited across the island of Ireland: Four in Northern Ireland via the Northern Ireland Clinical Research Network (Primary Care), and four in the Republic of Ireland by research team members. The practices were assigned as cluster sites to either the intervention or control arm (four per group) which were geographically similar and were stratified by jurisdiction, and further by rurality prior to randomization. Those GPs and PMs in the intervention group were invited to be interviewed about their experience with the intervention and its process of implementation. One practice declined to be interviewed (in NI) as they did not wish their information to be published, which left three GPs and three PMs to be interviewed, along with five research nurses, and the adjunct pharmacist.

### Interview schedule

A semi-structured interview schedule with open-ended questions and prompts, informed by the expertise of the research team [25, 26], was developed. Items included ‘How did the intervention work in your practice—can you tell me about the process?’; ‘Were there any barriers to implementing the intervention, or would there be any future barriers to doing so?’ and ‘What experience do you feel is necessary to fulfil the position of a research nurse on the ACP study?’; ‘What are your thoughts on the suitability of the home environment for meetings with patients?’; ‘Do you think the ACP model fits with the running of a GP practice’, and ‘Overall, were you satisfied or dissatisfied with the intervention or process—did you find it acceptable?’. Questions were supplemented with prompts where appropriate. Interviews took place face-to-face between October 2019 and February 2020.

### Ethical considerations

Ethical approval was obtained in the ROI from the Research Ethics Committee, Irish College of General Practitioners in January 2019 (reference ICGP2018.4.10). In NI, approval was received from the Office for Research Ethics, Northern Ireland (reference 19/NI/0001). All participants provided written, informed consent prior to interview.

### Data analysis

An expert research team organized and managed the data using the software NVivo-12 QSR International. The transcribed interview data were thematically analysed [19] in a bottom-up approach and generated an open and modifiable codebook. The CFIR [16] guided the analysis. Patterns, commonalities, and differences were identified and interpreted, leading to a theme structure for each ISH group and final thematic framework (see Table 1). Source triangulation (12 ISH/three professions, across two jurisdictions) and researcher triangulation (two researchers involved in data analysis) were employed in order to strengthen findings and improve rigour. Researchers observed reflexivity to minimize potential bias and influence. Pseudonyms (IDs) were used for participants to afford anonymity.

## Results

Important lessons were learnt from the feasibility study to take forward to a full clinical trial and to inform similar studies. A summary of findings is provided for each ISH group below, with Table 1 listing the main themes of each group in more detail, along with illustrative quotes, an indication of shared themes (‘S’) as well as mapping across to the five CFIR domains as applicable.

**Table 1:** Implementing stakeholders thematic framework

Implementing stakeholder group	Main themes	Shared themes	CFIR domain	Illustrative quotes	ID
General practitioners and practice managers	• Existing ACP efforts—beware of duplication: Medical Care Planning	S	OS	‘We are a practice that does a lot of Medical Care Planning. And it's structured in a way that you are remunerated for the work that you do.’ [ ] ‘We wouldn't be trying to organize anything extra.’	L3PM
	• Funding, staffing, and time are barriers		OS	‘I think it's the challenge of scaling it up in terms of funding and personnel.’ ‘The challenge would be the time. Time was the biggest challenge.’ ‘Unless there's greater funding going into social care, I really feel it's going to be very difficult to care for patients in their homes.’	L3GP F6GP L3GP
	• Participation was enjoyable as was made easy by study team		**PI**	‘So, from our point of view, from your side you’ve made it very easy. It hasn’t been a difficult one; it has been a good experience for us.’	L3GP
	• Trained ACP Nurses doing home visits facilitates ACP	S	IC/CIN	‘The home visit from the nurse was very good because as GPs, you know, we’re covering a big rural area.’ ‘As GPs we have limited time because we're also running acute surgeries and all the rest of what we do.’	F6GP L3GP
	• Medication review requires access to patient medical history to be effective	S	IC	‘I suppose when the drugs were reviewed; it was a pharmacist outside that was used, so they didn't really have the full clinical information about the patient. I suppose we have our own practice pharmacist now.’ ‘The medication review was good for sure.’	L3GP F6GP
	• Multidisciplinary teams are central to intervention		IC/CIN	‘I suppose we're mainly talking about the multidisciplinary team in order to make it happen.’	L3GP
	• Fragmentation of Services is a barrier		OS	‘Even though we all provide primary care, I'm isolated from the public health nurses and I’m also isolated from the physicians; OT’s. So that is a bit of an issue.’	EGP
Pharmacist	• Medication review should be done by pharmacist	S	IC	‘I think we’ve realized now that with really good care of the older patient, that each of the health care professionals has their own individual role. And where the nurses did a fantastic job, elucidating somebody’s drug history is not their speciality. That would be the role of the pharmacist.’	PHST
	• ACP is essential for older adults		IC	‘I think with the volume of patients and the workload that's already heaped upon GPs and the way that there's sort of a filter for information coming from secondary care, from specialist services, that it's very difficult to pay the attention to detail that's needed to provide a really good quality health care service for older patients. So, it's absolutely essential, I think, for the care of older people.’	PHST
	• Lack of access to patient data was a barrier		**PI**	‘The one thing that did impede the flow was the anonymizing of the information from the southern participants. So, each document that they sent me was password protected and redacted. I think it was a bit of an overkill, because the NI RNs just emailed me with the patient's confidential details omitted which worked completely fine. I couldn't identify the patient if I tried but, you know, the pertinent information is still there.’	PHST
	• Lack of access to past medical history was a barrier		**PI**	‘But the real problem that I faced was the lack of information and the information in situ. [ ] And I think if the study was to be redesigned, then the gold standard would be that the pharmacist would do their own medicines reconciliation or medicines review with the patients themselves and have access to all the information that I would have in my general routine in practice.’	PHST
	• Sustained constructive collaboration from the study team		**PI**	‘A huge thanks to the team, they were entirely inclusive and really open to all the sessions at every point along the way. It's been an absolute delight to work with them and it's been a really positive experience overall for me.’	PHST
	• Existing ACP efforts–beware of duplication: comprehensive geriatric assessment	S	OS	‘We would call it Comprehensive Geriatric Assessment and it would be in our team; it's consultant led and consultant geriatrician and he’d lead our team and undertake it to reach patients, and but then we have our full MDT.’	PHST
	• Multidisciplinary teams are central to ACP	S	IC/CIN	‘So, we understand the need for OT, physio and social work, and speech and language therapy, nursing staff. You know, everybody has their own clear and visible roles and it's all very important to the elderly.’	PHST
Nurses	• Appropriateness of PRISMA-7 for patient selection		**PI**	‘The patients fill out the Prisma-7 questionnaire, and that is a self-reported questionnaire of their frailty. And I didn't think that was terribly accurate.’ ‘A lot of the patients that we went out to see didn't have that many needs identified. And on numerous occasions we came out and thought how did they get in through the selection process?’	RN3 RN2
	• Training was beneficial		**PI**	‘I thought the training was very good. There were three days training and I enjoyed it a lot. I really did think it was it was comprehensive and delivered very, very well. ‘It was a bit more detailed than what we eventually ended up doing. But yes, I did feel it was great to have the talk from the pharmacist. You know, everybody had their part to do of their speciality. So, you got the benefit of all that.’	RN1 RN3
	• ACP home visits are key for best outcomes	S	IC	‘Certainly, for some patients, I don't think it could have been done anywhere else.’ ‘Yes, because you can get a lot of insight from them in terms of, you know, other hazards around; does the place look cared for? Do they look cared for? How they are mobilizing in their own environment and things like that.’ ‘I thought it was very appropriate, because there you got an insight into their housing conditions, into their social conditions.’	RN3 RN2 RN5
	• Procedural sequence facilitated the research nurses’ role		**PI**	‘There was a lot that we did like yeah! The training was very good. And then the follow through, we went out with [name] the pharmacist to see what her role was and the fact that [the patients] had already consented by the time we got out. The procedure of each step was well thought out.’	RN2
	• Patients appreciated the intervention		IS	‘And from, you know, speaking to the patients. They really appreciated it. Definitely. It could work really well for the ones who did need it.’	RN1
	• GP ideal setting but lack of time is barrier	S	IS	‘Well, GPs and practice nurses definitely would not have time for it. But the Federations now have practice-based pharmacists and social workers. So, there are other people, you know, in GP practices who might be able to help.’	RN1
	• Experienced nurse with specific skillset required: older adults, person-centred, chronic illness, health and social care system, community, and voluntary groups		IC/CIN	‘I think you possibly need the development of a GP primary care outreach nurse.’ ‘Somebody with experience of chronic illness and, because it is based in the community, an understanding of community services would be useful.’ ‘Knowing how to approach the older person, how to build that trusting relationship.’ ‘Having a good insight into community support that's available and what's available within the surgery and having good communication skills with the relevant GP and having a good system to fall back on, to get actions put into place.’	RN5 RN4 RN5 RN2
	• Lack of communication and protocols for data collection and recording was a barrier		**PI**	‘Just better communication from the study team throughout the study and better communication also between the nurses in the South and the nurses in the north.’ ‘I didn't feel at sea in any way and understood what had to be achieved in terms of the actual intervention. But I think I come back to my previous issue, which was knowing what data I should be collecting or capturing and whether I should be doing it in another way or different way.’	RN1 RN4
	• Multidisciplinary teams are central to ACP	S	IC/CIN	‘But I think that it needs to be taken on by somebody other. It's not a GP or practice nurses’ job to provision the time for it but all these new people now with the multi-disciplinary teams. Then I think, you know, you have some kind of mechanism there by which you could make it work.’	RN1

### Findings from GPs and practice managers

GPs’ and PMs’ responses were concerned with the ‘Inner Setting’ (IS) and ‘Intervention Characteristics’ (IC) domains of the CFIR, that is practical implications and systemic challenges, as well as resources and service fragmentation (Outer Setting (OS)), and characteristics of individuals involved (CIN), e.g., the necessity for multidisciplinary teams (see Table 1). However, in summary for this ISH group the preventative potential of the ACP intervention with its beneficial implications both in patient health terms as well as from a health systems perspective, was fully recognized and appreciated by GPs.

‘So rather than waiting for somebody to fall, have a fracture, or so many bad days, or have deteriorated and then us having to go to the house or the hospital or the ambulance having to go to the house, by doing this you can identify the at-risk patients, put the systems in place and then prevent something.’ (F6GP)

GPs and practice managers regarded the specially trained ACP nurses who completed the home visits, as core facilitators, not least because GPs have limited capacity to conduct such visits (F6GP, L3GP). The medication review was considered a beneficial component, although, in terms of implementation, the lack of access to patients’ medical history for the adjunct pharmacist was seen as a barrier. Integrated working with a multidisciplinary team at primary care level was considered an essential facilitator, and the importance of ensuring concurrent health and social care provisions was pointed out to ensure sustainable ACP support for patients in their own homes. Systemic barriers to incorporating the intervention long-term were identified as lack of funding, staffing levels, and time necessary to host the intervention, as well as fragmentation of health and social care services due to a lack of integrated working. There was a call for a clear structure in terms of process and funding (F6GP). It was highlighted that while funding will be required to support this primary care based intervention, its preventative nature was believed to have the potential to save much more money long-term.

‘So, I think it’s a good investment to put it into the primary care system.’ (F6GP)

In NI, GPs stressed the importance of avoiding duplication of existing efforts, e.g., the Medical Care Planning exercise (L3GP, L3PM), but perhaps finding ways of complementing, integrating with, and enhancing them. Participation in the study was enjoyable (F6GP), and not perceived as challenging but described as ‘very easy’ (L3GP) due to the efficiency of the research nurse.

### Findings from the adjunct pharmacist

The majority of the pharmacist’s responses focused on the ‘Intervention Characteristics’ (IC) and ‘Process of Implementation (PI) domains of the CFIR (see Table 1). In intervention terms, it was emphasized that ACP is vital for the care of older adults, and that multidisciplinary teams are imperative to provide efficient, effective, and sustainable ACP. In NI, the Comprehensive Geriatric Assessment was declared as an existing, consultant geriatrician led ACP exercise. As with the Medical Care Planning program cited by GPs, the pharmacist felt that there may be potential overlap with the ACP intervention.

In terms of the implementation process, there were two essential lessons learnt as to what is required to ensure that the pharmacist can provide a thorough and accurate review and optimization of patients’ medication. One is that the medication review should be conducted directly by the pharmacist, rather than by the ACP nurses with the pharmacist’s input; and the other that the pharmacist requires full access to patients’ medical history and data collated by the ACP nurses.

‘And where the nurses did a fantastic job, you know, elucidating somebody’s drug history, it's not their specialty. That would be the role of the pharmacist.’ (PHST)

The study team received praise for sustained constructive collaboration. An issue flagged by the adjunct pharmacist was of variations in patient data protection approaches. There were differences in handling data protection guidelines between GP practices which presented a barrier in terms of access to patient records, preventing direct access to the patients’ medical history, impeding informed decision-making.

### Findings from the research nurses

The largest volume of interview data resulted from the research nurses, the majority of which pertained to the ‘process of implementation’ (PI) domain of the CFIR (see Table 1). While some of their feedback was critical of aspects of the implementation process, it is essential to gain an understanding of these barriers in order to improve the process going forward. For example, the nurses stated that many patients appeared reasonably well and therefore wondered about the suitability of the screening tool used in patient selection [20] the parameters of which allowed for admission of a population of reasonable health.

Concerns were raised about ethical and procedural restrictions, following on from interpretation of the General Data Protection Regulation at the time of the study, which prevented them to actively carry out initial patient screening at GP practices in line with inclusion and exclusion criteria. Instead, screening had to be conducted by practice staff, and was therefore, subject to variation between practices (RN3). The research nurses (RN1, RN2) highlighted the importance of employing a patient assessment tool that reflected a biopsychosocial and person-centered approach, taking into account the personal circumstances of the individual patient, and their risk level of functional decline.

While the training supplied prior to intervention delivery was regarded as a facilitator in itself, insufficient communication within the study team during the implementation period was identified as a barrier to the implementation process (RN3). Going forward, care should be taken to clarify expectations, and to share pertinent information with research nurses in both jurisdictions timely and fully. In addition, the use of standardized protocols not only for data collection but also data recording was identified as a requirement to provide guidance to research nurses and ensure uniformly recorded data.

‘I think feeling not sure whether I’d collected the right information or knowing whether I should have done that differently. I think it would have been helpful to have some structure of what the expectations were.’ (RN4)

In terms of the intervention itself, the home visits were considered a key facilitator on which its success rested. These visits allowed for holistic assessment by providing necessary insight into how patients mobilize in their own environment, and what kind of support they have or need. The RNs also felt that the patients were also more comfortable in their own environment and thus more likely to disclose information (RN4).

It was stated that a newly created role for an ACP nurse was a key future requirement (RN5, RN3). Their skillset and expertise would include working with older adults, chronic illness, person-centered care, ability to build rapport with patients, knowledge, and expertise of navigating the health and social care system as well as the voluntary and community sector. At a systemic level the nurses regarded the primary care setting as ideal for the intervention but recognized that time allocation may be a problem for GPs, unless adequate resourcing is in place. GP Federations [27] in Northern Ireland, who could provide structure and direction, as well as multidisciplinary primary care teams working in an integrated fashion, were also considered facilitators (RN3).

Shared themes across participant groups included concerns around potential duplication with existing ACP efforts, and insufficient access to patient medical records. Multidisciplinary teams, home visits by specially trained nurses, and the medication review by a pharmacist were considered elementary components of the intervention.

## Discussion

We have interviewed the professional stakeholders involved in the implementation of the study to elicit their experience with and thoughts on the implementation process, and their views on the intervention itself. This will allow facilitators and barriers to implementation to be considered at the design stage of future testing of primary-cared based complex health interventions. The lessons we have learnt include helpful information about the intervention components and the implementation process. Guided by the CFIR [15], and as indicated within the feedback from each of the professional stakeholder groups, we have been able to identify ‘outer setting’, ‘inner setting’, ‘intervention characteristics, and ‘characteristics of the individuals involved’ components impacting on the ACP intervention which can now be addressed going forward and can be pre-empted by similar intervention efforts. Equally, we have learnt invaluable lessons in terms of the ‘process of implementation’ which should be taken forward to a full trial as well as applied to similar research, and applied interventions.

Firstly, findings showed that views on the primary-care based, nurse-led ACP intervention were favorable overall, with the caveat that existing efforts like Medical Care Planning and Comprehensive Geriatric Assessment are not duplicated. Lack of funding and staffing were seen as systemic barriers. The recommendations of the ISH have echoed those of patients and key health professionals (reported elsewhere, under review). In summary, the findings of this feasibility study indicate that an effective and sustainable ACP intervention may require the creation of a new, primary care-based nursing role, working with the support of GPs, PMs, and pharmacist, and with full access to a multidisciplinary team. It is envisaged that a full trial will provide full and clear guidance on appropriate implementation. The 2019 long term reform plans of the UK and Ireland [9, 10] have the potential to help provide the necessary infrastructure to introduce and sustain the ACP intervention albeit perhaps with regional adaptations. Efforts are required to ensure equity in access across both jurisdictions.

Home visits are considered the cornerstone of the intervention and essential to providing effective holistic care for older adults at risk of functional decline. The role of the pharmacist is an essential part of this holistic approach in providing medicine optimization collaboratively with the nurse and the GP, and with access to patients’ medical history. GP Federations in NI could provide structure and support, and ensure equity of access; however, similar umbrella organizations are not in existence in ROI at this point in time. Equally, while GP surgeries in NI often have access to pharmacists within their primary care teams, this is not the case in ROI.

Secondly, in terms of the implementation process, we have identified components which are necessary for best practice and outcomes. These include a structured approach with standardized procedures and protocols for study participant selection at appropriate level, data collection, data protection, and reporting; effective and sustained communication within and between roles and jurisdictions/regions, ensuring information is shared and everyone has an opportunity to input throughout; and application of best practice selection and assessment tools.

For future trans-jurisdictional primary care intervention studies, it would be pertinent to consider the differences in the primary care service models in NI and ROI, with the ROI having a mixed public-private structure. This might make supporting a healthcare intervention somewhat more challenging for GPs in ROI, and be potentially detrimental to equal patient access. If the intervention was scaled up it would require capacity and commitment from GPs, PMs, pharmacists, and designated ACP nurses. Collaboration with the voluntary and community sector may be helpful in facilitation at regional level.

To overcome barriers to implementation such as access to medical records, patient screening, and potential duplication in a full trial and beyond, they are currently being addressed as part of an impact acceleration project. Preparations for a full trial will include the development of standardized templates for recording patient information for use in both jurisdictions.

## Limitations

The strengths of the evaluation include its transparency and transferability. Limitations include that one GP practice in NI did not consent to be interviewed; their contribution may have yielded further data. Furthermore, this was a relatively small sample. If a larger, definitive cluster trial was conducted the sample would be larger and may yield additional themes.

## Implications

The lessons from the feasibility study have direct implications for informing a full trial, similar studies, and similar interventions. The challenges which presented themselves during the feasibility trial as highlighted by the ISH pertain to both the intervention itself as well as its implementation. If this intervention were to be upscaled the lessons gleaned from the feasibility study need to be applied both at systemic level (inner setting and outer setting) as well as at implementing team level (intervention characteristics, characteristics of individuals involved, process of implementation) to ensure effectiveness.

## Conclusion

Going forward, in a full trial efforts to improve the implementation process of this intervention should focus on employing standardized procedures and protocols that clarify and govern data protection, access to patient records, effective communication, screening, assessment, and uniform recording of research nurses’ data.

Our findings have helped to begin to carve out the characteristics of the role of the anticipatory care nurse, and the supporting infrastructure required to make this a feasible and sustainable primary care based ACP intervention.

## Supplementary Information

**Additional file 1:** COREQ Checklist.doc. Consolidated criteria for reporting qualitative studies (COREQ): 32-item checklist

**Additional file 2:** TIDieR Checklist.doc. The TIDieR (Template for Intervention Description and Replication) Checklist

## Data Availability

The data underlying this study cannot be shared publicly because they are qualitative patient interviews which contain personal and potentially identifiable information, and participants have consented to publication of anonymous quotes only. Requests for data can be made to Queen’s University Belfast Research Governance, Ethics and Integrity department (researchgovernance@qub.ac.uk) with appropriate ethical approval.

## Abbreviations

ACP: Anticipatory Care Plan
CFIR: Consolidated Framework for Advancing Implementation Research
CIN: Characteristics of the individual involved
cRCT: Cluster randomized controlled trial
GP: General practitioner
HSE: Health service executive
IC: Intervention characteristics
IS: Inner setting
ISH: Implementing stakeholders
NHS: National Health Service
NI: Northern Ireland
OS: Outer setting
PHN: Public health nurse
PHARM: Pharmacist
PI: Process of implementation
PM: Practice manager
RN: Research nurse
ROI: Republic of Ireland
