# Supplementary material for: Lessons from a feasibility study testing an anticipatory care planning intervention for older adults at risk of functional decline: feedback from implementing stakeholders
Source: Pilot Feasibility Stud. 2022 Jan 19;8:10. doi: 10.1186/s40814-022-00973-w (PMC8767659; doi:10.1186/s40814-022-00973-w)
Supplement: Supplementary file 1 — Additional file 1: COREQ Checklist.doc. Consolidated criteria for reporting qualitative studies (COREQ): 32-item checklist [file 40814_2022_973_MOESM1_ESM.docx]

**Additional File 1. Consolidated criteria for reporting qualitative studies (COREQ): 32-item checklist**

| Domain 1: Research team and reflexivity |  |
| --- | --- |
| Personal Characteristics |  |
| 1. Interviewer/facilitator Which author/s conducted the interview or focus group? | A non-authoring member of the research team, included in the Acknowledgements section. |
| 2. Credentials What were the researcher’s credentials? E.g. PhD, MD | PhD |
| 3. Occupation What was their occupation at the time of the study? | Research Fellow (RF) |
| 4. Gender Was the researcher male or female? | Female |
| Experience and training |  |
| 5. What experience or training did the researcher have? | Refer to TIDieR checklist |
| Relationship with participants |  |
| Relationship established |  |
| 6. Was a relationship established prior to study commencement? | No. |
| Participant knowledge of the  interviewer | |
| 7. What did the participants know about the researcher? e.g. personal goals, reasons for doing the research | They knew that RF was going to talk to them about their experience with, views of, and recommendations for, the ACP intervention. |
| Interviewer characteristics |  |
| 8. What characteristics were reported about the interviewer/facilitator? e.g. Bias, assumptions, reasons and interests in the research topic | Experienced, female researcher; observed reflexivity to minimize bias |
| Domain 2: study design |  |
| Theoretical framework |  |
| Methodological orientation and Theory |  |
| 9. What methodological orientation was stated to underpin the study? e.g. grounded theory,  discourse analysis, ethnography, phenomenology, content analysis | Thematic analysis |
| Participant selection |  |
| 10. Sampling. How were participants selected? e.g. purposive, convenience, consecutive, snowball | These were participating professional stakeholders in the study. |
| 11. Method of approach. How were participants approached? e.g. face-to-face, telephone, mail, email | A written brief and information by email, followed by a telephone call to arrange the interview. |
| 12. Sample size. How many participants were in the study? | There were 12 participating professional stakeholders interviewed for the current paper. |
| 13. Non-participation. How many people refused to participate or dropped out? Reasons? | One surgery (GP and PM) declined to be interviewed as they did not want their information to be published. |
| Setting |  |
| 14. Setting of data collection. Where was the data collected? e.g. home, clinic, workplace | Data was collected at participants’ work places. |
| 15. Presence of non-participants Was anyone else present besides the participants and researchers? | No. |
| 16. Description of sample. What are the important characteristics of the sample? e.g. demographic data, date | Participating GPs (3), Practice Managers (3), Adjunct Pharmacist (1), Research Nurses (5) |
| Data collection |  |
| 17. Interview guide. Were questions, prompts, guides provided by the authors? Was it pilot tested? | Yes, in the section ‘Interview Schedule’ in Methods. This study is the pilot. |
| 18. Repeat interviews Were repeat interviews carried out? If yes, how many? | No. |
| Audio/visual recording |  |
| 19. Did the research use audio or visual recording to collect the data? | Interviews were audio recorded and transcribed verbatim. |
| 20. Field notes. Were field notes made during and/or after the interview or focus group? | Yes. |
| 21. Duration. What was the duration of the interviews or focus group? | 30 minutes on average. |
| 22. Data saturation. Was data saturation discussed? | No. |
| 23. Transcripts returned. Were transcripts returned to participants for comment and/or correction? | No. |
| Domain 3: analysis and findings |  |
| Data analysis |  |
| Number of data coders |  |
| 24. How many data coders coded the data? | Two. |
| Description of the coding tree |  |
| 25. Did authors provide a description of the coding tree? | Yes. |
| Derivation of themes |  |
| 26. Were themes identified in advance or derived from the data? | Derived from the data in a bottom-up, inductive approach. |
| 27. Software. What software, if applicable, was used to manage the data? | NVivo-12. |
| 28. Participant checking. Did participants provide feedback on the findings? | No. |
| Reporting |  |
| 29. Quotations presented. Were participant quotations presented to illustrate the themes / findings? Was each  quotation identified? e.g. participant number | Yes. Participants IDs denote jurisdiction. |
| 30. Data and findings consistent. Was there consistency between the data presented and the findings? | Yes. |
| 31. Clarity of major themes. Were major themes clearly presented in the findings? | Yes. |
| 32 Clarity of minor themes. Is there a description of diverse cases or discussion of minor themes? | Yes. |
|  |  |

A. Tong et al.

Downloaded from https://academic.oup.com/intqhc/article/19/6/349/1791966 by guest on 14 September 2020
